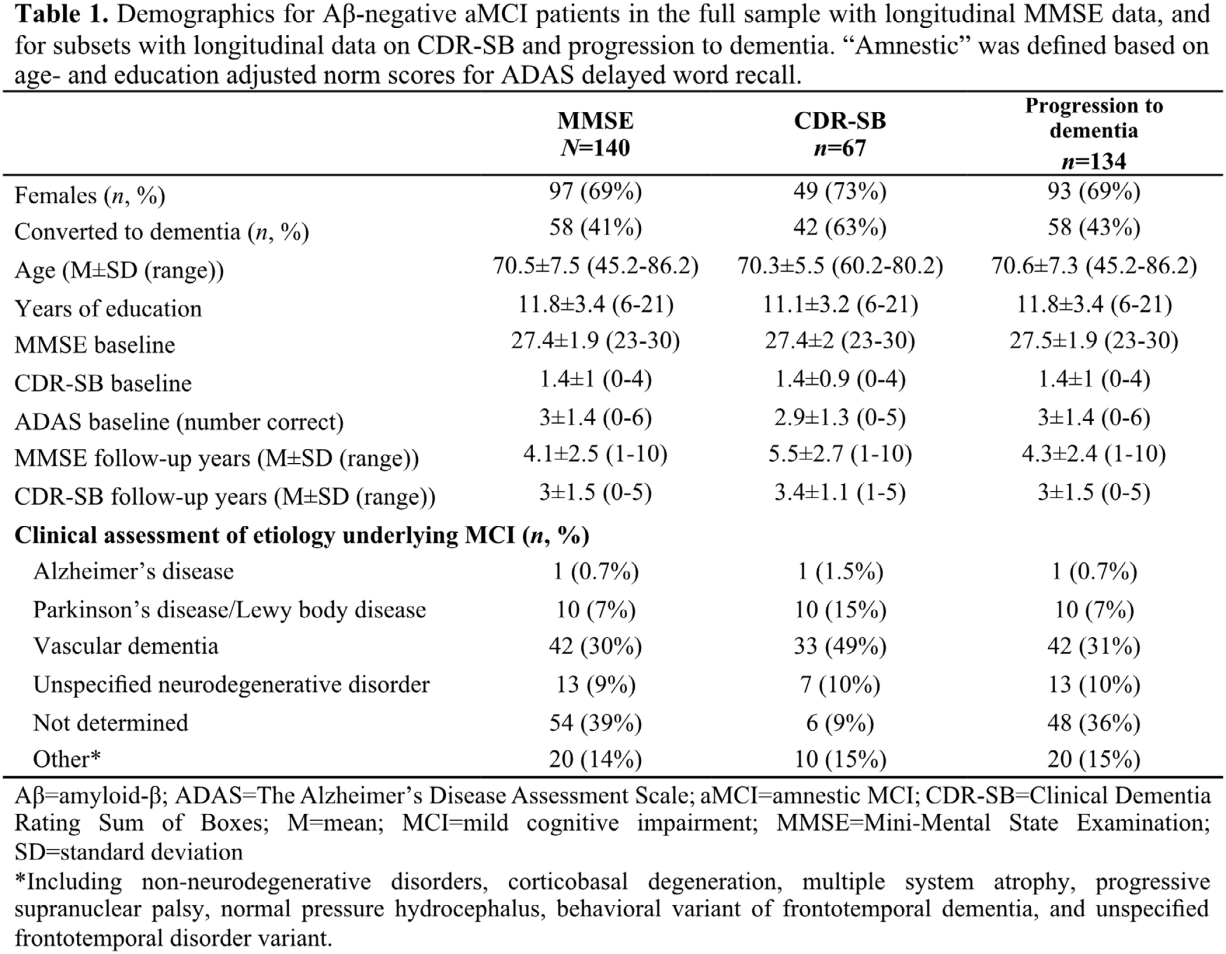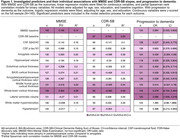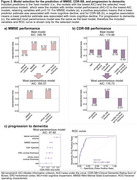# Predicting cognitive decline in amyloid‐negative individuals with amnestic mild cognitive impairment

**DOI:** 10.1002/alz70856_103071

**Published:** 2025-12-26

**Authors:** Amanda Annettesdotter, Nicola Spotorno, Anika Wuestefeld, Niklas Mattsson‐Carlgren, Olof Strandberg, Rik Ossenkoppele, Erik Stomrud, Sebastian Palmqvist, David A. Wolk, Oskar Hansson, Laura E.M. Wisse

**Affiliations:** ^1^ Department of Clinical Sciences Lund, Lund University, Lund, Sweden; ^2^ Clinical Memory Research Unit, Department of Clinical Sciences Malmö, Lund University, Lund, Sweden; ^3^ Clinical Memory Research Unit, Lund University, Lund, Sweden; ^4^ Clinical Memory Research Unit, Department of Clinical Sciences Malmö, Faculty of Medicine, Lund University, Lund, Sweden; ^5^ Memory Clinic, Skåne University Hospital, Malmö, Skåne, Sweden; ^6^ Center for Neurodegenerative Disease Research, Perelman School of Medicine, University of Pennsylvania, Philadelphia, PA, USA; ^7^ Department of Clinical Sciences Lund, Lund University, Lund, Lund, Sweden

## Abstract

**Background:**

A considerable portion of patients with amnestic mild cognitive impairment (aMCI) have negative amyloid‐β (Aβ) biomarkers and are therefore unlikely to have Alzheimer's disease (AD). Potential causes of cognitive decline in this heterogeneous group include limbic‐predominant age‐related TDP‐43 encephalopathy (LATE), cardio/cerebrovascular diseases, primary age‐related tauopathy (PART), and subthreshold Aβ. The prognosis of Aβ‐negative (Aβ‐) aMCI patients, putatively more benign, is unclear. We aim to investigate which predictors – including demographics, baseline cognition, fluid and imaging biomarkers – can best predict cognitive decline and progression to dementia in Aβ‐ aMCI.

**Method:**

We included 140 Aβ‐ aMCI patients (Aβ status based on cerebrospinal fluid (CSF) and positron emission tomography, when available; ‘amnestic’ based on norm scores for AD Assessment Scale delayed word recall) from BioFINDER‐1/2 with longitudinal Mini‐Mental State Examination (MMSE), and subsets with longitudinal data on Clinical Dementia Rating Sum of Boxes (CDR‐SB; *n* = 67) and progression to dementia (*n* = 134, 43% progressors; Table 1). Predictors included global and regional atrophy measures, specific for LATE and PART, CSF Aβ42/40 and *p*‐tau181, hypertension, white matter hyperintensities and global cognition. Individual MMSE and CDR‐SB slopes were estimated with linear mixed‐effects models. Associations of predictors with MMSE/CDR‐SB slopes and progression to dementia were tested. Significant predictors and demographic variables were included in the model selection process using R package MuMIn, which tests linear combinations of variables and ranks models by the Akaike information criterion (AIC).

**Results:**

Figure 1 shows individual associations for the identification of significant predictors for the model selection process. For MMSE (AIC: 350.57; Figure 2a), the selected most parsimonious model included baseline MMSE and whole‐brain cortical thickness. For CDR‐SB (AIC: 176.73; Figure 2b), baseline CDR‐SB, amygdala volume, and middle frontal gyrus cortical thickness were included. For progression to dementia (AIC: 97.65; Figure 2c), the selected model included MMSE, lateral ventricles volume, entorhinal and whole‐brain cortical thickness, sex and CSF Aβ42/40.

**Conclusions:**

Baseline cognition, global and regional atrophy measures are valuable predictors of cognitive decline in Aβ‐ aMCI, with regional brain measures hinting at specific pathologies. These prediction models are relevant for the new LATE clinical criteria. We aim to validate our findings in ADNI.